# Immune-cell imbalance and immunometabolic reprogramming in chronic prostatitis/chronic pelvic pain syndrome

**DOI:** 10.3389/fimmu.2026.1926409

**Published:** 2026-10-02

**Authors:** Hao Li, Andong Cheng, Mengfan Wang, Shiyang Zhou, Xixi Wang, Xingchi Zhu, Kun Wang, Yu Guan, Jialin Meng, Chaozhao Liang

**Affiliations:** 1 Department of Urology, The First Affiliated Hospital of Anhui Medical University, Hefei, China; 2 Institute of Urology, Anhui Medical University, Hefei, China; 3 Anhui Province Key Laboratory of Urological and Andrological Diseases Research and Medical Transformation, Anhui Medical University, Hefei, China; 4 First School of Clinical Medicine, Anhui Medical University, Hefei, China; 5 School of Life Sciences, Anhui Medical University, Hefei, China

**Keywords:** immunometabolic reprogramming, macrophage polarization, neuro-immune crosstalk, NLRP3 inflammasome, precision medicine

## Abstract

Available evidence suggests that disruption of immune homeostasis may contribute to chronic prostatitis/chronic pelvic pain syndrome (CP/CPPS) beyond the effects of localized infection. This narrative review uses immune cells as its organizing axis and synthesizes recent evidence from single-cell sequencing, Mendelian randomization, and multi-omics. The available findings suggest that the M1/M2 dichotomy may not fully capture macrophage heterogeneity in prostatic tissues and that activation of the NLRP3 inflammasome and IL-1β release may form a candidate link between innate immunity and the Th17 response. On this basis, the review discusses how glycolytic and oxidative-stress-related reprogramming may shape immune-cell states; considers chemokine networks such as CCL5/CCR5 and CXCL10/CXCR3 in the recruitment of macrophages, Th1 cells, and Th17 cells to prostatic tissue; and examines how mast cells and neurogenic inflammation may participate in the bidirectional neuro-immune dialogue associated with persistent pain. At the intervention level, we distinguish human clinical observations and interventions from findings in cell systems and animal models, and discuss candidate strategies involving the NLRP3 inflammasome, chemokine receptors, microbiota-derived metabolites, neuromodulation, and local or multi-component approaches. These concepts require prospective validation in clinically defined CP/CPPS subgroups before they can support mechanism-guided treatment.

## Introduction

Chronic prostatitis/chronic pelvic pain syndrome (CP/CPPS) is a complex clinical syndrome centered on persistent pelvic pain or discomfort, frequently accompanied by lower urinary tract symptoms and sexual dysfunction, severely impairing the quality of life of young- and middle-aged men. Its global prevalence remains high, constituting a substantial public-health burden (1). Although its etiology and pathogenesis have not been fully elucidated, immune-inflammatory responses are increasingly implicated in disease onset and persistence (2, 3).

In recent years, research has moved beyond the traditional infection-inflammation dichotomy to focus on the dynamic imbalance of the local prostatic immune microenvironment, particularly the complex crosstalk between innate and adaptive immunity and the chronic inflammation and neural sensitization it produces (4). Detailed dissection of this network is essential for breaking the diagnostic and therapeutic bottlenecks of CP/CPPS (5, 6). Current research has already sketched the key nodes and networks of the immune-inflammatory response in CP/CPPS. The subsequent sections of this review will sequentially unfold the core topics of macrophage polarization imbalance (7), NLRP3 inflammasome activation (8, 9), immunometabolic reprogramming (10), chemokine-mediated recruitment (10), and neuro-immune crosstalk (4, 11).

However, significant knowledge gaps and controversies remain. First, the classical M1/M2 dichotomy is no longer sufficient to describe the high heterogeneity of macrophages under disease conditions, and the origin, regulation, and precise role of disease-specific subpopulations in pain generation remain unclear (7). Second, novel cell-death modalities of immune cells, such as ferroptosis and pyroptosis, in CP/CPPS have only recently been revealed; how they interact with classical inflammatory pathways and together amplify tissue damage to form a vicious cycle of unresolvable chronic inflammation is an urgent scientific question (12, 13). Moreover, the maintenance mechanisms of chronic pelvic pain—the core symptom of CP/CPPS—cannot be fully explained by local inflammation alone, and effective interventional strategies targeting neuro-immune circuits remain very limited. Finally, and most challenging, is the marked clinical heterogeneity of CP/CPPS. Patients show vastly different responses to the same treatment, suggesting different underlying immune endotypes or molecular subtypes. Although Mendelian-randomization studies suggest causal relationships between specific circulating immune-cell phenotypes and disease risk (14), a precision molecular-typing system based on the local prostatic immune microenvironment that could directly guide clinical practice has not yet been established.

Accordingly, this narrative review synthesizes recent advances in the immune-inflammatory mechanisms of CP/CPPS, focusing on macrophage polarization, NLRP3 inflammasome activation, immunometabolic reprogramming, chemokine networks, novel cell-death modalities, and neuro-immune crosstalk. We organize these findings into a multilevel, dynamically interconnected model (**Figure 1**) and critically consider limitations in mechanistic depth, technical approaches, model relevance, and clinical translation.

**Figure 1:**
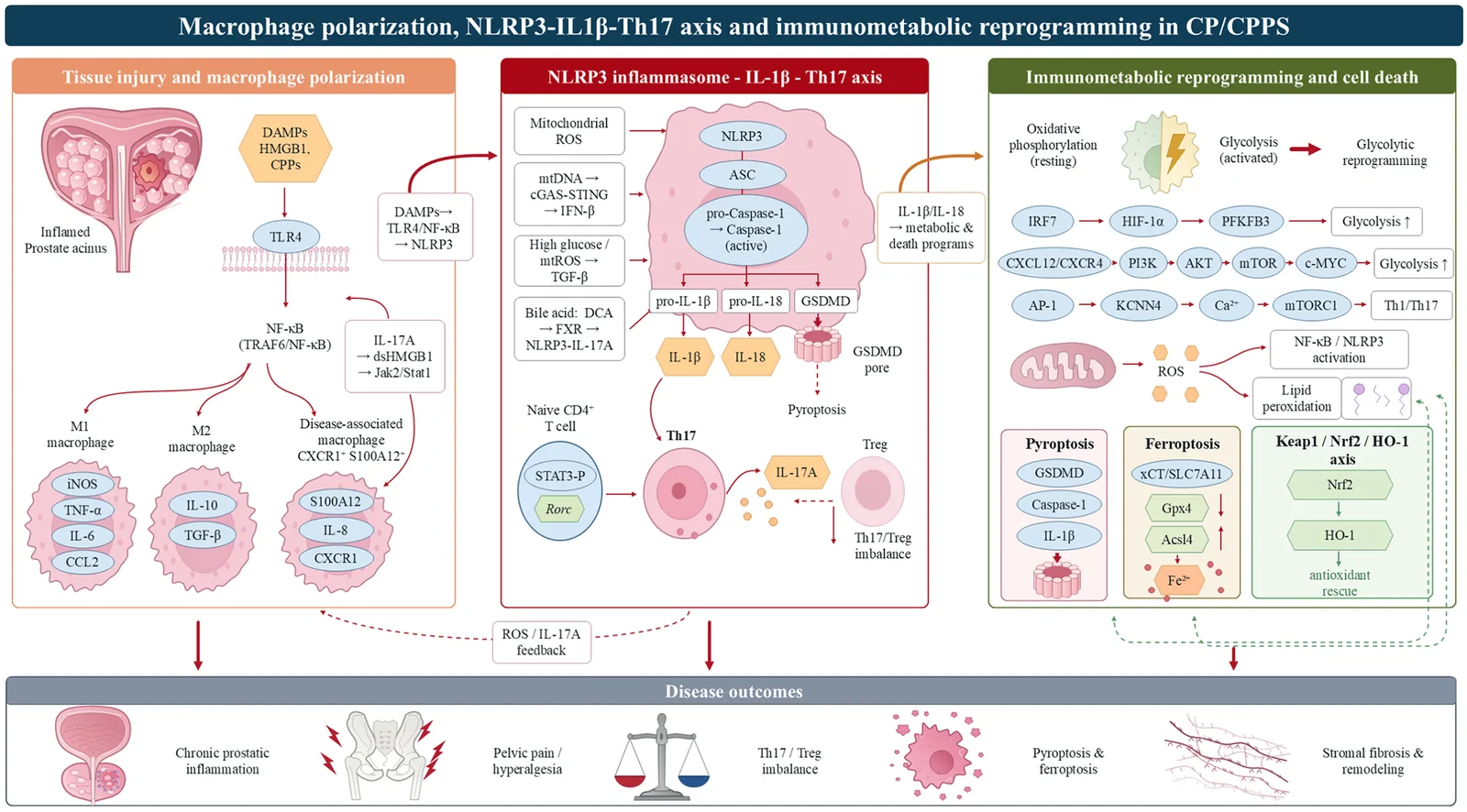
Proposed immune–inflammatory cascade in CP/CPPS: macrophage polarization, the NLRP3–IL-1β–Th17 axis and immunometabolic reprogramming. In the prostatic immune microenvironment of CP/CPPS, tissue-injury–derived DAMPs such as HMGB1 engage TLR4–TRAF6–NF-κB signaling to drive CD11b^+^iNOS^+^ M1 polarization and the secretion of TNF-α, IL-6 and CCL2; single-cell sequencing additionally identifies a CXCR1^+^ S100A12^+^ disease-associated macrophage subset whose abundance correlates with pelvic-pain scores. Mitochondrial ROS, mtDNA–cGAS–STING signaling, high-glucose–TGF-β crosstalk and gut-microbiota perturbations (e.g. loss of deoxycholic acid, FXR repression) converge on NLRP3 inflammasome assembly; Caspase-1 cleavage of pro-IL-1β/pro-IL-18 and GSDMD-mediated pyroptosis amplify innate inflammation, while secreted IL-1β phosphorylates STAT3 to skew naive CD4^+^ T cells and Tregs toward Th17. Th17-derived IL-17A feeds back through dsHMGB1/Jak2/Stat1 to entrench M1 polarization. In parallel, three glycolytic axes—IRF7→HIF-1α→PFKFB3, PI3K/AKT/mTOR/c-MYC and AP-1/KCNN4/mTORC1—drive metabolic reprogramming, with mitochondrial ROS simultaneously triggering NF-κB/NLRP3 activation and lipid peroxidation. xCT/GPX4 down-regulation and ACSL4 up-regulation precipitate ferroptosis, which together with pyroptosis forms a self-amplifying metabolism–cell death loop, while the Keap1/Nrf2/HO-1 pathway represents an endogenous antioxidant rescue. The cascade converges on chronic prostatic inflammation, pelvic hyperalgesia, Th17/Treg imbalance, pyroptosis and ferroptosis, and stromal fibrosis as disease end-points.

## Macrophage polarization imbalance: from the M1/M2 dichotomy to disease-specific subpopulations

Macrophages in prostatic tissue can no longer be fully encompassed by the classical M1/M2 framework. In the chronic inflammatory microenvironment of CP/CPPS, macrophages adapt to constantly changing local signals through a continuous spectrum of phenotypic polarization, forming multiple disease-specific subpopulations that both drive persistent inflammation and potentially participate in tissue repair and immune tolerance. Understanding the dysregulation of M1/M2 balance and its downstream molecular events may help clarify mechanisms of immune dysregulation. This section starts from the classical M1/M2 roles, then examines the discovery of disease-specific subpopulations and the key molecular signals driving polarization imbalance.

### The pro-inflammatory role of M1 macrophages

In the pathological progression of CP/CPPS, excessive activation of M1 macrophages may contribute substantially to tissue inflammation and pain. Studies in the experimental autoimmune prostatitis (EAP) model show that macrophages infiltrating the prostatic tissue polarize markedly toward the pro-inflammatory M1 phenotype, characterized by high expression of inducible nitric oxide synthase (iNOS) and abundant secretion of inflammatory mediators such as tumor necrosis factor-α (TNF-α) and interleukin-6 (IL-6) (15). High-mobility group box 1 (HMGB1), as a key damage-associated molecular pattern, is upregulated in CP/CPPS and, by enhancing TRAF6-mediated activation of the nuclear factor kappa B (NF-κB) pathway, directly drives the polarization of CD11b^+^iNOS^+^ M1 macrophages, thereby exacerbating prostatic inflammation and hyperalgesia (16). In addition, peroxiredoxin 5 (Prdx5) is upregulated in M1 macrophages in a reactive-oxygen-species (ROS)-dependent manner and, by activating the Toll-like receptor 4 (TLR4)/NF-κB pathway, further amplifies M1 polarization signals and promotes prostatic epithelial cell apoptosis and disease progression (17). These pro-inflammatory macrophages not only release inflammatory factors themselves but also recruit additional immune cells by secreting chemokines such as CCL2, forming a positive-feedback loop that maintains the chronic inflammatory state (18). In autoimmune disease patients, macrophage-derived TNF-α not only drives inflammation but also stimulates prostatic fibroblast proliferation, promoting the development of benign prostatic hyperplasia (BPH); TNF blockade significantly reduces epithelial hyperplasia, NF-κB activation, and macrophage-mediated inflammation in prostatic tissue (19). In addition, calciprotein particles (CPPs), as physiologically relevant inducers of mineral stress, can effectively induce pro-inflammatory activation of monocytes, upregulating a range of pro-inflammatory genes and cytokines including IL-6, CXCL8, CCL2, and macrophage migration inhibitory factor (MIF), providing a new perspective on the inflammatory pathology associated with calcium homeostasis imbalance (20).

### Functional complexity of M2 macrophages

In contrast to the pro-inflammatory M1 phenotype, M2 macrophages, which carry anti-inflammatory and tissue-repair functions, display functional complexity in the pathological process of CP/CPPS. Traditionally, M2 macrophages have been thought to promote inflammation resolution and tissue repair by secreting factors such as interleukin-10 (IL-10) and transforming growth factor-β (TGF-β). However, in the chronic inflammatory microenvironment, M2 macrophage function may shift or become dysregulated. Studies have found that Eriocalyxin B treatment of an EAP model promotes macrophage conversion from M1 to M2 and is accompanied by altered gut microbiota and elevated vitamin D3 levels, with the latter shown to promote M2 polarization *in vitro*, suggesting an important role for the microbiota-metabolite axis in regulating macrophage phenotypic switching (21). Excessive activation of M2 macrophages may also be related to the fibrotic process. In CP/CPPS, sustained inflammatory stimulation may lead M2 macrophages to secrete pro-fibrotic factors such as TGF-β, thereby promoting prostatic stromal collagen deposition and tissue remodeling—one possible mechanism by which the disease becomes chronic and progresses toward BPH. Thus, M2 macrophages in CP/CPPS are not simple protectors; their functional state is highly dependent on the dynamic balance of the local microenvironment. Mendelian-randomization studies further support a role for immunoregulatory factors in benign prostatic disease, with elevated circulating levels of interleukin-10 receptor subunit α (IL-10RA) showing a suggestive association with increased prostatitis risk (22).

### Discovery of novel disease-associated macrophage subpopulations

Going beyond the classical M1/M2 dichotomy, cutting-edge technologies such as single-cell transcriptomics are revealing high macrophage heterogeneity in the prostatic microenvironment of CP/CPPS. Marc et al. found that an S100A12-expressing CXCR1^+^ myeloid cell population is closely associated with IL-8-mediated inflammation. The alarmin S100A12 secreted by this cell population was significantly positively correlated with patients’ pain scores, suggesting that it may represent a specific pathogenic subpopulation involved in neuro-immune interactions and in the pelvic pain and dysfunction associated with CP/CPPS (23). Chen et al. used cytokine-array screening and found that CCL5 was the most markedly upregulated chemokine in samples from patients with CP/CPPS. This chemokine drives the expansion of a CCR5^+^ macrophage subset and, through the CCL5/CCR5/SHP2 axis, inhibits SHP2-mediated dephosphorylation of STAT1, thereby sustaining NF-κB activation and locking macrophages into a pro-inflammatory state (24). Zhang et al. found that a CXCR4^+^ macrophage subset undergoes glycolytic metabolic reprogramming through the PI3K/AKT/mTOR–c-MYC–PFKFB3 cascade, thereby driving M1 polarization and prostatic fibrosis. Silencing CXCR4 restored mitochondrial function, promoted the transition from M1 to M2 polarization, and reduced collagen deposition (25). Meng et al. identified an Irf7+ macrophage subset in an EAP model using RNA-seq and demonstrated that Irf7, as a transcription factor, directly binds to the Hif-1α promoter, thereby driving glycolytic metabolic reprogramming and sustaining M1 polarization and stromal fibrosis (10). Hong et al. reported a Kir2.1^+^/TRPV2^+^ macrophage subset with sensitivity to electrical signaling. They showed that 4-Hz transcutaneous electrical nerve stimulation induces membrane depolarization by downregulating Kir2.1 and reduces Ca²^+^ influx, thereby shifting intracellular signaling from the NF-κB/STAT1 pathway toward STAT6 activation. This process promotes M1-to-M2 repolarization and alleviates pelvic pain (26). Another single-cell sequencing study of peripheral blood immune cells in CP/CPPS patients, although mainly focused on CD8^+^ TEMRA cells, also provided a technical framework for in-depth analysis of myeloid subpopulations, hinting at the possible existence of additional functionally specialized macrophage or monocyte-derived subpopulations under disease conditions (27). The transcriptional profiles, surface markers, and functional features of these newly identified disease-associated subpopulations cannot be summarized by simple M1 or M2 labels; they represent cellular states that have evolved within specific niches of chronic inflammation, with unique pro-inflammatory or regulatory functions, providing a new molecular basis for precision-targeted therapy. For example, in BPH tissue, high-grade inflammation is significantly associated with elevated IL-8 and IL-6 levels, suggesting that specific inflammatory microenvironments may shape unique immune-cell subpopulations (28).

### Upstream signals of macrophage polarization

Macrophage phenotypic polarization is precisely regulated by a complex upstream signaling network. The chemokine axis plays a key role; in addition, signals from other immune cells are also important. For example, MIF released by CD4^+^ T cells can promote inflammation by activating the PI3K/AKT pathway, while IL-17A produced by Th17 cells can induce prostatic epithelial cells to release double-stranded HMGB1 (dsHMGB1), which in turn drives macrophage M1 polarization via the Jak2/Stat1 pathway, forming a vicious cell-cell loop (29). These multilevel upstream signals together build a complex regulatory network that determines the ultimate fate and functional output of macrophages in the CP/CPPS microenvironment. Studies have also found that heterozygous deletion of E-cadherin exacerbates macrophage inflammation and stromal hyperplasia in the aged-mouse prostate, indicating that changes in cell-adhesion molecules are also important upstream factors regulating the local immune microenvironment (30) (**Table 1**).

**Table 1:** Mechanistic nodes and precision-intervention targets of immune dysregulation in CP/CPPS, organized by category.

Category	Target/node	Principal cell type	Mechanistic role	Effect of perturbation/intervention	References
Macrophage polarization	HMGB1/TLR4/NF-κB	M1 macrophage	HMGB1 enhances TRAF6-mediated NF-κB signaling, driving CD11b^+^iNOS^+^ M1 polarization and the release of TNF-α/IL-6/CCL2	HMGB1 neutralization or TLR4/NF-κB inhibition (e.g. radiofrequency hyperthermia, electroacupuncture) reduces prostatic inflammation and hyperalgesia	(16, 36, 98)
Macrophage polarization	Prdx5	M1 macrophage	Up-regulated in a ROS-dependent manner; amplifies M1 polarization through TLR4/NF-κB	Targeting Prdx5/ROS dampens prostatic epithelial apoptosis and disease progression	(17)
Macrophage polarization	IL-10/TGF-β (M2 axis)	M2 macrophage	Resolution and tissue repair; under chronic stimulation TGF-β output skews towards fibrosis	Eriocalyxin B, vitamin D3 and microbiota-derived metabolites promote M2 polarization, but BPH-like fibrotic drift must be monitored	(21, 101)
Macrophage polarization	CXCR1^+^ S100A12^+^ subset	Disease-associated myeloid cells	Single-cell-defined disease-associated subset secreting S100A12/IL-8; abundance correlates with pelvic pain score	Candidate stratification marker and target for the neuro-immune pain axis	(23)
NLRP3 inflammasome	NLRP3/ASC/Caspase-1	M1 macrophage, prostatic epithelium	Senses ROS, mtDNA and bile-acid (DCA) loss as PAMPs/DAMPs; assembles the inflammasome and cleaves pro-IL-1β/IL-18	Juglone, ursolic acid and Xialiqi capsule directly inhibit the NLRP3-Caspase-1-GSDMD axis	(31–35, 77–79)
NLRP3 inflammasome	GSDMD (pyroptosis)	Macrophage, epithelium	Caspase-1 cleaves GSDMD, forming a plasma-membrane pore that mediates inflammatory pyroptosis and releases DAMPs	pH/ROS dual-responsive dexamethasone nanoformulations suppress pyroptosis-derived mediators and relieve symptoms	(42, 77, 79)
NLRP3 inflammasome	IL-1β/STAT3/Th17	CD4^+^ T cells, Treg	IL-1β phosphorylates STAT3 to drive naive CD4^+^ T cells and Tregs toward a Th17 phenotype, causing Th17/Treg imbalance	Isoliquiritigenin and Resolvin D1 inhibit NLRP3/IL-1β via Nrf2/HO-1, restoring Th17/Treg balance	(37–39)
Immunometabolic reprogramming	IRF7 → HIF-1α/PFKFB3	M1 macrophage	Up-regulates the glycolytic programme, driving pro-inflammatory polarization and prostatic fibrosis	Blocking IRF7-HIF-1α or PFKFB3 promotes M1 → M2 phenotypic switching	(10, 25)
Immunometabolic reprogramming	PI3K/AKT/mTOR/c-MYC	Macrophage, Th1/Th17	Alcohol and CXCL12/CXCR4 signals converge on this axis to amplify glycolysis, c-MYC and Th1/Th17 expansion	LY294002 (PI3K inhibitor) or alcohol cessation attenuate EAP inflammation and pelvic pain	(25, 46, 61)
Immunometabolic reprogramming	AP-1/KCNN4/mTORC1	CD4^+^ T cells	Enhances Ca²^+^ signaling and activates mTORC1, promoting Th1/Th17 differentiation	Ca²^+^-activated K^+^ channel blockade represents a candidate T-cell metabolic checkpoint	(48)
Immunometabolic reprogramming	Keap1/Nrf2/HO-1	Macrophage, epithelium	Master antioxidant switch; loss of activity allows ROS accumulation and parallel amplification of NLRP3 and ferroptosis	Astaxanthin, astragaloside IV and sodium butyrate engage Nrf2/HO-1 to inhibit NLRP3 and ferroptosis and ameliorate fibrosis	(43, 50, 88, 89)
Novel cell death	xCT/GPX4/ACSL4 (ferroptosis)	Prostatic epithelium, immune cells	In EAP, down-regulated xCT-GPX4 and up-regulated ACSL4 with iron overload converge on ferroptosis	Deferoxamine and astragaloside IV partially reverse inflammation, fibrosis and mast-cell activation	(31, 43)
Chemokine network	CCL5/CCR5/STAT1	Macrophage, Th17	Drives M1 polarization through STAT1 phosphorylation and forms a positive-feedback loop with MIF and IL-17A/CCL20	CCR5 blockade or MIF antagonists disrupt the self-reinforcing macrophage-Th17 circuit	(24, 57, 58)
Chemokine network	CXCL10/CXCR3/PI3K-AKT	Th1, spinal macrophages	Drives Th1 differentiation and migration; spinal-cord CXCL10 sustains central sensitization via JAK/STAT3	AMG487 reduces Th1 differentiation, suppresses M1 macrophages and relieves EAP pelvic pain	(11, 61, 62)
Chemokine network	CXCL12/CXCR4	T cells, macrophages	Engages AKT/NF-κB and STAT3 to sustain inflammatory infiltration	AMD3100 substantially reduces infiltration in chronic non-bacterial prostatitis	(25, 67)
Chemokine network	CCL2/MCP-1 (incl. exosomal miR-203a-3p)	Monocytes/macrophages	Recruits monocytes; epithelium-derived exosomes carry miR-203a-3p that up-regulates stromal MCP-1	Ningmitai capsule lowers CCL2 and inhibits NF-κB/STAT3, relieving pelvic pain	(64, 65, 82)
Neuro-immune crosstalk	Mast-cell tryptase/PAR2	Mast cell, sensory neuron	mMCP7-like tryptases activate PAR2 and trigger pelvic tactile allodynia	Cromolyn plus cetirizine markedly lowers NIH-CPSI scores and reduces immune infiltration	(69, 70)
Neuro-immune crosstalk	SP/CGRP (neuropeptides)	Sensory neuron, mast cell, macrophage	Neuropeptides trigger mast-cell degranulation and amplify neurogenic inflammation	Cannabidiol delivers dose-dependent analgesia through CB2 and TRPV1 desensitization	(69, 70, 109)
Neuro-immune crosstalk	IL-1β → PVN NMDA-NR1	Central neurons	Prostate-derived IL-1β crosses the blood-brain barrier and up-regulates PVN NMDA-NR1, mediating behavioral changes	Intra-PVN IL-1β inhibition prolongs ejaculation latency and lowers NR1 expression	(72)
Gut microbiota/metabolites	Propionate/butyrate/GPR43/HDAC6	Th17/Treg	Short-chain fatty acids regulate Th17/Treg balance via GPR43 and HDAC6	Oral sodium butyrate activates Nrf2/HO-1, inhibits NLRP3 and relieves pelvic pain	(52, 88)
Gut microbiota/metabolites	DHA → PPARγ/NF-κB-IL-17A	Th17	Dietary DHA suppresses NF-κB/IL-17A through PPARγ, reducing the Th17 fraction	Provides a nutritional-metabolism-based adjunctive target	(87)
Gut microbiota/metabolites	5-HIAA → AhR/Sgk1/FoxO1	Th17	High-salt diet lowers 5-HIAA, derepressing Sgk1 and inactivating FoxO1, which favours Th17 differentiation	5-HIAA supplementation activates AhR and reverses Th17 skewing	(54)
Precision therapeutic strategies	Electroacupuncture/TENS/LIPUS	Neuro-immune interface	Inhibits P2X7R/NLRP3, TLR4/NF-κB and PI3K-AKT/JAK-STAT pathways	LIPUS at 1.0 W/cm² for 4 weeks restores Th17/Treg balance and down-regulates IL-1β/IL1R1/MyD88	(26, 36, 40, 97, 106, 108)
Precision therapeutic strategies	Local nanodelivery (HA/LF @ EMO Gel)	Prostatic macrophages	Crosses the blood-prostate barrier, accumulates in glandular lumens and is taken up by macrophages to modulate TLR4/NF-κB	Combined anti-inflammatory, anti-oxidant and anti-fibrotic action; a paradigm for local precision delivery	(95)

CP/CPPS, chronic prostatitis/chronic pelvic pain syndrome; EAP, experimental autoimmune prostatitis; BPH, benign prostatic hyperplasia; DAMP, damage-associated molecular pattern; PAMP, pathogen-associated molecular pattern; HMGB1, high-mobility group box 1; TLR4, Toll-like receptor 4; NF-κB, nuclear factor κB; Prdx5, peroxiredoxin-5; ROS, reactive oxygen species; NLRP3, NLR-family pyrin-domain-containing 3; ASC, apoptosis-associated speck-like protein; GSDMD, gasdermin D; IL, interleukin; STAT, signal transducer and activator of transcription; TGF-β, transforming growth factor β; iNOS, inducible nitric-oxide synthase; HIF-1α, hypoxia-inducible factor-1α; IRF7, interferon regulatory factor 7; PFKFB3, 6-phosphofructo-2-kinase/fructose-2,6-biphosphatase 3; mTORC1, mechanistic target of rapamycin complex 1; AP-1, activator protein-1; Keap1, Kelch-like ECH-associated protein 1; Nrf2, nuclear factor erythroid 2-related factor 2; HO-1, heme oxygenase-1; GPX4, glutathione peroxidase 4; ACSL4, acyl-CoA synthetase long-chain family member 4; xCT/SLC7A11, cystine/glutamate antiporter; CCL, C-C motif chemokine ligand; CCR, C-C motif chemokine receptor; CXCL, C-X-C motif chemokine ligand; CXCR, C-X-C motif chemokine receptor; MIF, macrophage migration inhibitory factor; MCP-1, monocyte chemoattractant protein-1; mMCP, mouse mast-cell protease; PAR2, protease-activated receptor 2; TRPV1, transient receptor potential vanilloid 1; CB2, cannabinoid receptor 2; SP, substance P; CGRP, calcitonin gene-related peptide; PVN, paraventricular nucleus; NMDA, N-methyl-D-aspartate; NR1, NMDA receptor subunit 1; SCFA, short-chain fatty acid; GPR43, G-protein-coupled receptor 43; HDAC6, histone deacetylase 6; PPARγ, peroxisome proliferator-activated receptor γ; AhR, aryl hydrocarbon receptor; Sgk1, serum/glucocorticoid-regulated kinase 1; FoxO1, forkhead box protein O1; TENS, transcutaneous electrical nerve stimulation; LIPUS, low-intensity pulsed ultrasound; NIH-CPSI, National Institutes of Health Chronic Prostatitis Symptom Index; HA, hyaluronic acid; LF, lactoferrin; EMO, baicalein.

## NLRP3 inflammasome activation and links to Th17 differentiation

The NLRP3 inflammasome is a protein complex that may link local prostatic tissue-damage signals with downstream adaptive immune activation. It senses pathogen-associated and damage-associated molecular patterns, activates caspase-1 to cleave pro-IL-1β/IL-18, and triggers gasdermin D (GSDMD)-mediated pyroptosis, releasing pro-inflammatory cytokines and additional danger signals that may amplify inflammation. IL-1β can promote naive CD4+ T-cell skewing toward Th17 in experimental systems, making the NLRP3 inflammasome a candidate link between innate and adaptive immunity. This section outlines the components and activation of the inflammasome, the IL-1β-mediated pathway of Th17 differentiation, and its intersection with programmed-cell-death modalities.

### Activation, components, and regulation of the NLRP3 inflammasome

Activation of the NLRP3 inflammasome is a key molecular event linking tissue damage with innate immune responses in CP/CPPS. Its activation is finely regulated by multiple upstream signals, among which oxidative stress and mitochondrial dysfunction are the core triggers. In the EAP model, prostatic tissue exhibits markedly increased lipid peroxidation products and decreased antioxidant enzyme activity, accompanied by abnormally elevated iron concentrations; this oxidative-stress environment provides ideal conditions for NLRP3 inflammasome assembly (31). In sleep-deprivation-induced EAP models, ROS accumulation has been shown to damage mitochondria and induce mitochondrial DNA release, thereby activating the cGAS–STING pathway, promoting interferon-β production, recruiting inflammatory cells, and shaping a pro-inflammatory microenvironment (32). In addition, high glucose intake has been shown to activate transforming growth factor-β through a mitochondrial ROS-dependent pathway, thereby promoting NLRP3 inflammasome activation (33). Beyond metabolic stress, specific microbial metabolites also participate in regulation. For example, antibiotic treatment alters the gut microbiota and reduces levels of the secondary bile acid deoxycholic acid; deoxycholic acid then suppresses farnesoid X receptor (FXR) expression and activates the NLRP3-IL-17A axis, promoting Th17-cell differentiation (34). These studies together reveal that NLRP3 inflammasome activation is a multilevel process integrating metabolic abnormalities, oxidative damage, and microbial-dysbiosis signals. Early-life exposure to environmental endocrine disruptors combined with a high-fat diet can programmatically induce NLRP3 inflammasome activation and IL-1β upregulation in the adult prostate, providing a new perspective for understanding the early origins of adult CP (35). In addition, in a rat model of chronic non-bacterial prostatitis (CNP), electroacupuncture has been shown to alleviate inflammation by inhibiting the TLR4/NF-κB signaling pathway and downregulating nuclear translocation of NF-κB p65, suggesting that TLR4 signaling may serve as one of the upstream regulatory nodes of NLRP3 (36).

### Potential role of the NLRP3-IL-1β axis in Th17 differentiation

The activated NLRP3 inflammasome generates active caspase-1 by cleaving pro-caspase-1, which in turn processes the inactive pro-IL-1β and pro-IL-18 into their mature forms; among these, IL-1β release can contribute to Th17-cell differentiation. In the EAP model, NLRP3 inflammasome activation is significantly enhanced, IL-1β levels rise in prostatic tissue and serum, and the magnitude of this rise correlates positively with disruption of the Th17/Treg balance (37). Mechanistic studies show that IL-1β can effectively promote naive CD4^+^ T-cell differentiation toward Th17 cells *in vitro* and enhance the conversion of regulatory T cells (Tregs) to Th17 cells, mediated by promoting phosphorylation of signal transducer and activator of transcription 3 (STAT3) (37). The role of this axis has been confirmed in reverse through multiple intervention strategies. For example, isoliquiritigenin inhibits oxidative stress and NLRP3 inflammasome activation by activating the nuclear factor erythroid 2-related factor 2 (Nrf2)/heme oxygenase-1 (HO-1) signaling pathway, thereby reducing IL-1β release and attenuating Th17-mediated inflammation (38). Similarly, Resolvin D1 also inhibits the NLRP3 inflammasome through the same Nrf2/HO-1 pathway, ameliorating inflammation and pain in the EAP model (39). Together, these findings support the NLRP3-IL-1β axis as a candidate link through which innate immune signals may influence adaptive immunity, particularly the Th17 response. Further studies have found that electroacupuncture treatment of chronic pelvic pain syndrome in rats can suppress the P2X7R/NLRP3 signaling pathway, downregulate caspase-1 and IL-18 expression, and thereby reduce the release of inflammatory factors, producing an anti-inflammatory and analgesic effect (40). In addition, bidirectional Mendelian-randomization analyses reveal potential causal relationships between circulating inflammatory cytokines and prostatitis, identifying IL-12B as a protective factor and IL-10RA as a risk factor, providing genetic evidence for the causal role of the cytokine network in disease (41) (**Table 1**).

### Intersections of the NLRP3 inflammasome with novel cell-death modalities

Within the complex inflammatory network of CP/CPPS, NLRP3 inflammasome activation is not an isolated event; it crosstalks closely with novel programmed-cell-death modalities such as pyroptosis and ferroptosis, jointly amplifying inflammatory damage. Pyroptosis is an inflammatory cell death triggered after caspase-1 cleaves GSDMD and is itself a direct downstream event of NLRP3 inflammasome activation. A study developed a pH/ROS dual-responsive dexamethasone nanoformulation that releases its payload in an acidic or ROS-rich microenvironment and is efficiently internalized by lipopolysaccharide-stimulated macrophages, alleviating symptoms by downregulating pro-inflammatory factor levels, with mechanisms partially involving regulation of the balance between cell death and inflammation (42). On the other hand, ferroptosis, an iron-dependent form of cell death characterized by lipid peroxidation, may also reinforce NLRP3 inflammasome activation. In EAP models, ferroptotic changes, including downregulation of the system Xc−/glutathione peroxidase 4 (GPX4) axis and upregulation of acyl-CoA synthetase long-chain family member 4 (ACSL4), have been observed. Notably, the iron chelator deferoxamine partially alleviates inflammation, fibrosis, and mast-cell activation (31). Research on astragaloside IV further links these two pathways, demonstrating that it inhibits ferroptosis by activating the Keap1/Nrf2/HO-1 axis, thereby alleviating the pathological damage of type III prostatitis (43). These interactions indicate that therapies targeting the NLRP3 inflammasome may need to simultaneously consider their impact on related cell-death pathways to achieve more comprehensive anti-inflammatory effects (**Figure 1**). In addition, other forms of cell death also participate. For example, dihydroartemisinin inhibits cell inflammation and proliferation in CNP by blocking the E2F7/HIF1α pathway (44). Exosomes secreted by calycosin-treated prostatic stromal cells attenuate inflammatory damage and apoptosis of prostatic epithelial cells by inhibiting the NF-κB signaling pathway and the expression of the apoptosis executor cleaved-caspase-3 (45).

## Immunometabolic reprogramming: glycolysis, oxidative stress, and cell death

The functional fate of immune cells is determined by their metabolic state. In CP/CPPS, both macrophage polarization toward M1 and Th17 cell polarization depend on a metabolic switch from oxidative phosphorylation to glycolysis, while mitochondrial dysfunction-induced ROS bursts further amplify inflammatory and cell-death signals. Glycolysis, oxidative stress, and cell death therefore form a coupled metabolic-inflammatory axis—a candidate disease mechanism and a potential source of druggable metabolic checkpoints. This section starts from glycolysis-driven polarization mechanisms and sequentially examines oxidative-stress reprogramming and the therapeutic potential of metabolic checkpoints.

### Glycolysis: M1 macrophage/Th17 polarization

In CP/CPPS models, immune-cell metabolic reprogramming may contribute to functional activation, with enhanced glycolysis reported as a metabolic feature of M1 macrophage and Th17-cell activation. Studies have shown that alcohol intake significantly exacerbates inflammation in the EAP model and promotes Th1-cell differentiation by activating the PI3K/AKT/mTOR pathway, and inhibition of this pathway alleviates inflammation, revealing the regulatory role of metabolic pathways in T-cell fate (46). Interferon regulatory factor 7 (Irf7) directly upregulates transcription of hypoxia-inducible factor-1α (Hif-1α), enhancing macrophage glycolytic programming and driving its polarization toward the pro-inflammatory M1 phenotype, thereby exacerbating prostatic inflammation and fibrosis (10). Activation of the CXCL12/CXCR4 axis similarly upregulates the key glycolytic enzyme PFKFB3 via downstream PI3K/AKT/mTOR/c-MYC signaling, promoting macrophage glycolysis and M1 polarization; blockade of this axis can shift macrophages toward the anti-inflammatory M2 phenotype (25). In addition, insulin-like growth factor 1 (IGF1) activates protein kinase C-β (PKC-β) via its receptor IGF1R, promoting Th17 differentiation and exacerbating prostatic inflammation, providing direct evidence that metabolic reprogramming influences T-cell fate (47). In a rat model of chronic non-bacterial prostatitis, enhanced activator protein-1 (AP-1) in CD4^+^ T cells transcriptionally activates KCNN4 expression, enhancing Ca^2+^ signaling and activating mTORC1, thereby promoting Th1/Th17 differentiation (48). Together, these studies indicate that glycolytic pathway activation may be a candidate metabolic link between upstream environmental signals and downstream pro-inflammatory immune-cell phenotypes.

### Oxidative stress drives immunometabolic reprogramming

Oxidative stress plays a dual role in the immunometabolic reprogramming of CP/CPPS, acting both as an important signaling molecule that activates immune cells and as a key effector factor causing tissue damage. The NADPH-oxidase inhibitor apocynin alleviates oxidative stress, suppresses androgen receptor (AR)-mediated cell proliferation and the TGF-β/NOX4 signaling pathway in a BPH model, and promotes anti-inflammatory programming of macrophages, indicating that targeting oxidative-stress sources has potential to regulate immune-cell function therapeutically (49). Astaxanthin reduces pro-inflammatory cytokine levels and alleviates oxidative-stress damage in a CP/CPPS rat model by inhibiting the NF-κB signaling pathway (50). Similarly, astragaloside IV improves oxidative-stress markers, inflammation, and fibrosis in CP/CPPS rats by activating the Keap1/Nrf2/HO-1 antioxidant pathway (43). Transcriptomic analyses further support the key role of oxidative stress in linking CP/CPPS and erectile dysfunction, with ROS metabolism as the key bridging interaction mechanism and the hub genes NFE2L2 and NQO1 involved (51). These findings reveal that oxidative stress is not merely a consequence of inflammation but a core metabolic node driving abnormal immune-cell activation and tissue damage; targeting antioxidant pathways provides a new therapeutic direction for restoring immunometabolic homeostasis.

### Therapeutic potential of metabolic checkpoints

Gut-microbiota-derived metabolites such as short-chain fatty acids (SCFAs) and bile acids, as key immunometabolic signaling molecules, profoundly influence CP/CPPS disease progression by directly or indirectly regulating immune-cell differentiation and function. Studies have found that gut-microbiota metabolite propionate levels are decreased in EAP mice; propionate supplementation regulates the Th17/Treg differentiation balance via its receptor GPR43 and histone deacetylase 6 (HDAC6), decreasing disease susceptibility (52). Similarly, Lycium barbarum polysaccharide inhibits Th17 differentiation by regulating the gut-microbiota metabolite putrescine, with mechanisms involving TRAF6-mediated inhibition of the JAK2-STAT3 pathway and consequent reduction of inflammatory factor synthesis (53). A high-salt diet exacerbates EAP by reducing beneficial gut microbiota and their metabolite 5-hydroxyindoleacetic acid (5-HIAA); supplementation with 5-HIAA activates the aryl hydrocarbon receptor (AhR), suppresses transcription of serum and glucocorticoid-regulated kinase 1 (Sgk1), restores forkhead box O1 (FoxO1) function, and thereby inhibits Th17-cell differentiation (54). In addition, alcohol intake alters the gut microbiota and metabolome of EAP mice, leading to a marked rise in the cholesterol metabolite 27-hydroxycholesterol, which promotes Th17 differentiation via a sterol regulatory element-binding protein 2 (SREBP2)-dependent pathway, revealing the role of the microbiota-cholesterol-metabolism axis in disease (55). Metabolomic analysis also found that EAP with comorbid depression is associated with changes in pathways such as the reductive acetyl-CoA pathway and L-lysine fermentation generating acetate and butyrate, with these changes regulated by specific bacterial genera (56). These pieces of evidence suggest that specific gut-microbiota metabolites constitute a key bridge linking environmental factors with local prostatic immune inflammation, precisely programming the fate of T cells and macrophages by acting on metabolic sensors or epigenetic modifiers within immune cells.

## Chemokine networks and immune-cell recruitment

For immune cells to properly home, reside, and function within prostatic tissue, they depend on a set of spatially specific chemokine-receptor pairings. CCL5/CCR5 dominates macrophage and Th17-cell recruitment and pro-inflammatory polarization, CXCL10/CXCR3 directs Th1-cell chemotaxis and localization to inflammatory foci, and other chemokine axes together shape the microscale spatial distribution and temporal dynamics of immune cells within tissue. Understanding this spatial-temporal network is the anatomical and molecular prerequisite for knowing where, when, and what immune cells do, and provides clear targets for receptor-blockade-based precision interventions (**Figure 2**). This section sequentially dissects the mechanisms and synergistic effects of the three dominant axes.

**Figure 2:**
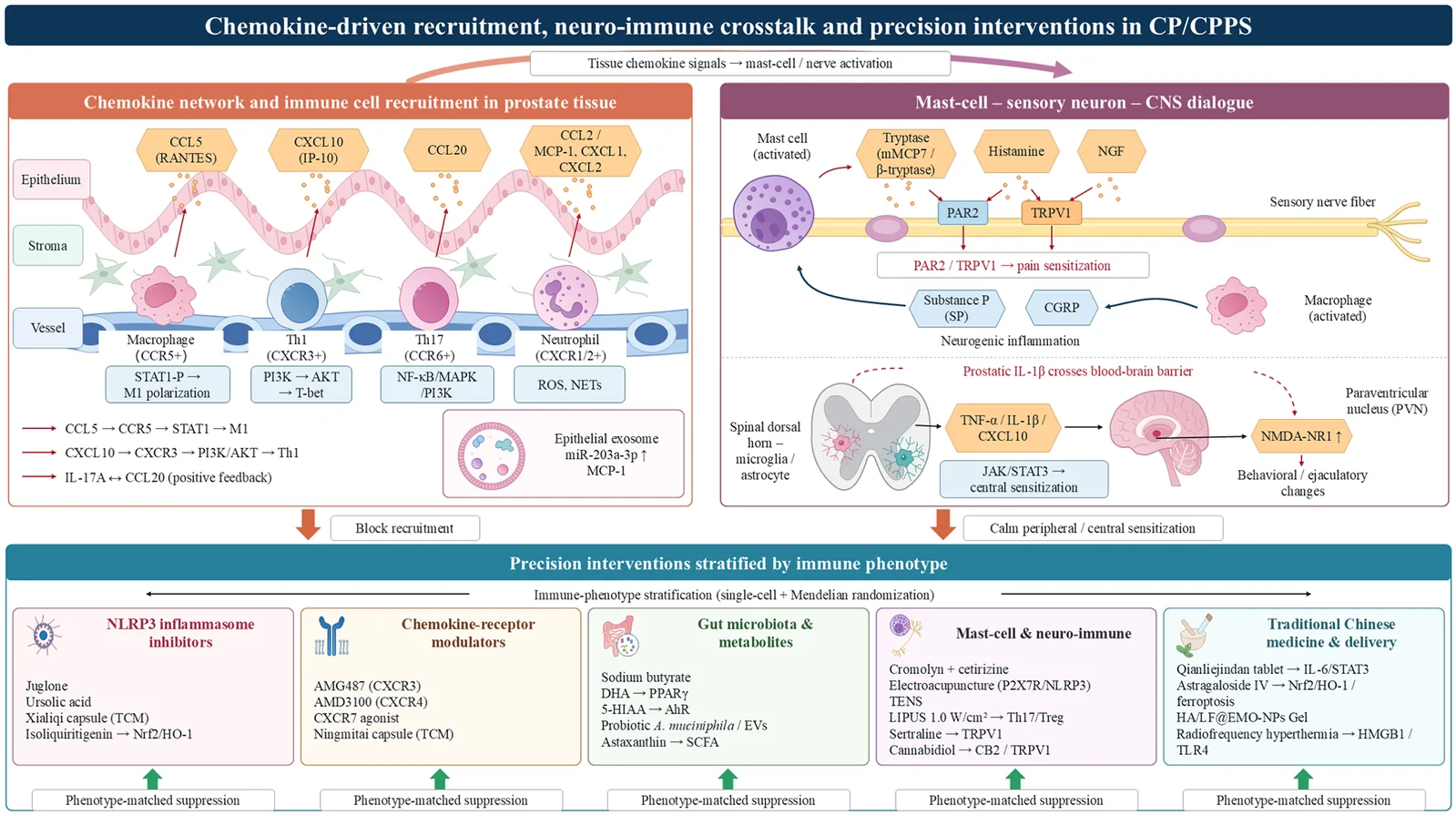
Tissue-level integration in CP/CPPS: chemokine-driven recruitment, mast-cell/neuro-immune crosstalk and candidate intervention targets. At the tissue level, a spatially restricted chemokine matrix coordinates immune-cell trafficking into the prostate: CCL5/CCR5 (STAT1-driven M1 polarization), CXCL10/CXCR3 (PI3K/AKT-dependent Th1 trafficking), and CCL2/MCP-1, CXCL1, CXCL2 and CCL20 collectively recruit M1 macrophages, Th1, Th17 cells and neutrophils into the prostatic stroma; epithelial exosomes loaded with miR-203a-3p further up-regulate MCP-1 to amplify monocyte ingress. In peri-glandular nerve fibers, activated mast cells release mMCP7-like tryptase, histamine and NGF that engage PAR2 and TRPV1 on sensory terminals to drive nociceptor sensitization, while sensory neurons reciprocally release substance P and CGRP that act back on mast cells and macrophages, constituting a bidirectional neuro-immune dialogue. Prostate-derived IL-1β crosses the blood–brain barrier to up-regulate NMDA-NR1 in the hypothalamic paraventricular nucleus (mediating behavioral changes), while microglia and astrocytes in the spinal dorsal horn sustain central sensitization through CXCL10 and the JAK/STAT3 pathway. Building on this tissue circuit, the review groups candidate interventions into five mechanistically defined modules: (i) NLRP3 inflammasome inhibitors (juglone, ursolic acid, Xialiqi capsule, isoliquiritigenin–Nrf2/HO-1); (ii) chemokine-receptor modulators (AMG487 for CXCR3, AMD3100 for CXCR4, CXCR7 agonists, Ningmitai capsule); (iii) gut-microbiota and metabolite-based therapies (sodium butyrate, DHA–PPARγ, 5-HIAA–AhR, A. muciniphila probiotics and extracellular vesicles, astaxanthin via SCFA); (iv) mast-cell and neuro-immune approaches (cromolyn plus cetirizine, electroacupuncture targeting P2X7R/NLRP3, TENS, LIPUS at 1.0 W/cm², sertraline acting on TRPV1, cannabidiol via CB2 and TRPV1); and (v) traditional Chinese medicine and local delivery (Qianliejindan tablet via IL-6/STAT3, astragaloside IV via Nrf2/HO-1 with anti-ferroptotic action, HA/LF@EMO-NPs Gel for rectal delivery, radiofrequency hyperthermia down-regulating HMGB1/TLR4).

### The CCL5/CCR5 axis drives macrophage polarization and Th17 differentiation

The chemokine axis formed by CCL5 (RANTES) and its main receptor CCR5 plays a core regulatory role in the immunopathology of CP/CPPS, far beyond simple cell chemotaxis. Studies have found that CCL5/CCR5 signaling directly drives macrophage polarization toward the pro-inflammatory M1 phenotype by activating signal transducer and activator of transcription 1 (Stat1) and inhibiting its dephosphorylation, thereby exacerbating local prostatic inflammation (24). This process synergizes with the classical NF-κB pathway, forming a positive-feedback loop that continually amplifies inflammatory signaling. More importantly, this axis is closely linked to Th17-cell differentiation. Treatment with macrophage migration inhibitory factor (MIF) significantly increases the numbers of CD4^+^IL-17A^+^ and CD4^+^RORγt^+^ Th17 cells in the EAP model, while MIF antagonists or anti-CD74 neutralizing antibodies reverse this effect, with mechanisms involving activation of the p38 MAPK pathway (57). This indicates that the inflammatory microenvironment driven by signals such as CCL5/CCR5 can further program T-cell fate through mediators such as MIF, promoting Th17 differentiation. In addition, IL-17A released by Th17 cells can stimulate macrophages to produce CCL20 via NF-κB/MAPK/PI3K pathways, and CCL20 is a key chemokine attracting Th17 cells, thereby forming a self-reinforcing vicious cycle between macrophages and Th17 cells and adding to the complexity and therapeutic difficulty of CP/CPPS (58). Interaction between hyaluronic acid (HA) and CD44 can likewise promote Th1-cell differentiation by stabilizing ANX A1 protein and activating the Akt/mTOR pathway, affecting disease susceptibility (59). In an autoimmune prostatitis model, CD8 T-cell deficiency did not alter the disease course, confirming that CD4-Th1/Th17-related immune responses are the key drivers of inflammation and chronic pelvic pain (60).

### Role of the CXCL10/CXCR3 axis in Th1 differentiation and migration

The chemokine receptor axis of CXCL10 (IP-10) and CXCR3 is a key pathway driving the Th1 immune response in CP/CPPS. In the EAP model, activation of the CXCL10/CXCR3 axis increases the proportion of Th1 cells and is accompanied by upregulation of PI3K/AKT signaling (61). The CXCR3-specific antagonist AMG487 and the PI3K inhibitor LY294002 effectively suppress CXCL10-mediated Th1-cell differentiation and migration, confirming the molecular mechanism by which this axis acts through the PI3K/AKT pathway (61). *In vivo* treatment with AMG487 not only improves inflammation and pelvic pain in EAP mice but also reduces Th1-cell differentiation by inhibiting the IL-12/STAT4 pathway and suppresses pro-inflammatory M1 macrophage activation through the lipopolysaccharide/NF-κB p65 pathway, thereby reducing the secretion of inflammatory mediators (62). This finding extends the role of the CXCL10/CXCR3 axis from simple T-cell chemotaxis to direct regulation of macrophage phenotype. The role of CXCL10/CXCR3 is not limited to the prostate locally. In CP/CPPS models, CXCL10 derived from dorsal-horn neurons and astrocytes is associated with spinal macrophage recruitment, and the axis mediates neuroinflammation by regulating the JAK/STAT3 pathway, providing a new perspective for understanding central sensitization and the maintenance of chronic pain (11). In urological chronic pelvic pain syndrome (UCPPS), upregulation of serine protease inhibitor B2 (SerpinB2) is associated with increased expression of the pro-inflammatory chemokines CXCR3 and CXCL10, suggesting that this axis has broad relevance in pelvic pain disorders more generally (63).

### Chemokine networks and intratissue spatial localization

The precise positioning and infiltration of immune cells within prostatic tissue depends on a complex, cooperative chemokine network in which CCL5/CCR5 and CXCL10/CXCR3 act together with other chemokines. Monocyte chemoattractant protein-1 (MCP-1/CCL2) is a key factor recruiting monocytes/macrophages to inflammatory sites; its expression is markedly upregulated in CP/CPPS and correlates with inflammation severity (64). In CP/CPPS, exosomes released by inflammatory prostatic epithelial cells are enriched in miR-203a-3p and promote inflammation of prostatic stromal cells by upregulating MCP-1 expression, revealing the role of intercellular communication in regulating the chemokine network (65). In addition, CXCL1 and CXCL2 are key downstream factors mediating IL-17-driven neutrophil chemotaxis; the use of IL-17 neutralizing antibodies or neutrophil depletion both ameliorates inflammation and hyperalgesia in EAP, indicating that the Th17-chemokine-neutrophil axis is an important link in the effector phase of inflammation (66). The CXCL12/CXCR4 axis also plays a role in chronic non-bacterial prostatitis: prostate-infiltrating inflammatory T cells highly express CXCR4, and its antagonist AMD3100 significantly reduces inflammatory infiltration via mechanisms involving inhibition of AKT/NF-κB and STAT3 signaling (67). These chemokine axes likely act as an interconnected network, coordinating immune-cell recruitment across disease stages and shaping the inflammatory niche of CP/CPPS. Urinary proteomics analysis further supports this view, finding that differentially expressed proteins in CPPS patients are enriched in biological processes such as reduced immune-cell migration (68).

## Neuro-immune crosstalk: mast cells and neurogenic inflammation

The pain features of CP/CPPS go far beyond what classical immune inflammation can explain, indicating deep involvement of the nervous system. As key effectors of neuro-immune coupling in prostatic tissue, mast cells directly sensitize sensory nerve terminals through release of histamine, tryptase, nerve growth factor and other mediators; neuropeptides, in turn, act backward from nerve terminals on immune cells, forming a bidirectional dialogue and positive feedback. This neurogenic inflammatory mechanism not only drives pain chronification but also provides a mechanistic basis for stratified neuro-immune combination therapy. This section starts from direct evidence for mast cells as pain effectors, then analyses neuropeptide-immune interactions and the molecular mechanisms of bidirectional coupling.

### Direct evidence for mast cells as pain effectors

Mast cells are key direct effector cells in the neuro-immune interaction of chronic pelvic pain (CPP); their activation and degranulation are core links connecting immune inflammation with neural sensitization. Clinical studies have found that in CP/CPPS patients, the active tryptase concentration in post-prostatic-massage urine (≥ 30 µg/mL) can identify a mast-cell-dysfunction subgroup; after targeted therapy with sodium cromoglycate and cetirizine, not only does the active tryptase concentration drop significantly but the NIH-CPSI total and subscores also improve markedly, accompanied by reduced abundance of CD45^+^ cells, macrophages, and neutrophils, indicating that mast-cell-targeted therapy can effectively relieve symptoms and modulate the immune-inflammatory environment (69). Animal models provide further mechanistic evidence. In a CP1-induced mouse model, activated tryptase-positive mast cells in the prostate of NOD/ShiLtJ mice are positioned closer to nerve fibers and express the mouse ortholog mMCP7; direct urethral instillation of mMCP7 (but not the β-tryptase ortholog mMCP6) induces acute pelvic tactile allodynia in C57BL/6J mice, and *in vitro* experiments confirm that mMCP7 induces cultured primary neurons to differentially activate pain- and inflammation-related molecules, directly demonstrating an important role for δ-tryptase in mediating the development of pelvic tactile allodynia (70). In addition, psychological stress in a novel mouse model not only induces mild prostatic inflammation but also significantly aggravates chemically induced prostatic inflammation, manifested as increased total numbers and activated proportion of mast cells, and simultaneously causes sustained bladder overactivity and pelvic hyperalgesia, revealing a biopsychosocial mechanism by which psychological factors participate in pain maintenance through effects on immune cells such as mast cells (71).

### Neuropeptide-induced immune-cell activation

Macrophages are not only the main drivers of local prostatic inflammation; the inflammatory mediators they release can also cross tissue barriers and affect the peripheral and central nervous systems, amplifying and chronifying pain signals. In EAP rats, IL-1β levels are significantly elevated in the prostate, serum, and cerebrospinal fluid, indicating that IL-1β is a key prostate-derived inflammatory cytokine capable of crossing the blood-brain barrier and affecting the central nervous system. Paraventricular nucleus microinjection of an IL-1β inhibitor in EAP rats gradually prolongs ejaculation latency and decreases NMDA NR1 subunit expression, while microinjection of IL-1β in control rats produces the opposite effect, confirming that IL-1β mediates pain-related behavioral changes by upregulating NMDA receptor expression in the paraventricular nucleus (72). In a rat chronic prostatitis model, inhibition of dorsal-horn microglia activation or the NOTCH1 receptor significantly decreases TNF-α and IL-1β content in the dorsal horn and reverses the enhanced electrical excitability of neurons, providing direct targets for intervening in central sensitization to relieve refractory pain (73). Clinical imaging studies also lend support: in CP/CPPS patients, functional connectivity strength between the posterior cingulate cortex and the left hippocampus correlates significantly with serum IL-6 levels, preliminarily revealing the link between brain functional changes and low-grade systemic inflammation and providing a new direction for understanding how cerebral functional circuits relate to clinical symptoms and systemic inflammation (74). In addition, in the EAP model, exogenous kallikrein, through a bradykinin B2 receptor-dependent pathway, inhibits the infiltration of inflammatory cells such as macrophages into the prostate and ameliorates glandular atrophy and fibrosis, suggesting that regulating macrophage function may be beneficial for relieving neuropathic-pain-related tissue remodeling (75).

### Bidirectional dialogue between nerves and immune cells

Active bidirectional communication occurs between neuropeptides released by sensory nerve terminals and immune cells, jointly shaping the microenvironment of chronic pelvic pain. Neuropeptides such as substance P (SP) and calcitonin gene-related peptide (CGRP) released by injured or activated sensory neurons can act directly on corresponding receptors on mast cells and macrophages, inducing degranulation or activation and the release of mediators such as histamine, tryptase, and TNF-α, which further sensitize nerve terminals and form a positive-feedback loop of neurogenic inflammation. Although the literature surveyed here does not directly include mechanistic studies on the role of SP or CGRP in CP/CPPS, related clinical observations indirectly support the existence of this interaction. In addition, proteases such as tryptase released after mast-cell activation can also cleave and activate protease-activated receptors (PARs) on sensory neurons, such as PAR2, directly generating pain signals. This neuropeptide-immune-cell axis constitutes a highly localized and rapidly responsive alarm system that, in CP/CPPS, may become hyperactive due to sustained low-grade inflammation, leading to persistent reductions in pain threshold and hyperalgesia (**Figure 2**). Clinical studies have also found that volunteers with chronic prostatitis-like symptoms have a significantly higher incidence of premature ejaculation, suggesting that altered nerve function is closely related to symptoms, even though specific cytokine and transcription-factor levels in their semen did not differ (76).

## Candidate precision immune interventions for CP/CPPS

The following candidate interventions are discussed with the study design and disease model specified for each example.

### Candidate strategies targeting the NLRP3 inflammasome

NLRP3 inflammasome signaling has emerged as a candidate therapeutic target in CP/CPPS. In an EAP mouse model and a macrophage-pyroptosis cell model, juglone was reported to bind to and suppress key proteins such as NLRP3, caspase-1, and GSDMD, effectively suppressing macrophage pyroptosis and alleviating oxidative stress in animal and cell models, thereby relieving EAP symptoms; the effect is reversed by the NLRP3 agonist BzATP (77). In an *in vitro* model of lipopolysaccharide- and ATP-induced pyroptosis in RWPE-2 prostate epithelial cells, medicated serum of Xialiqi capsules significantly improves cell viability, reduces ultrastructural features of pyroptosis, and decreases supernatant IL-18 and nitric oxide levels, with mechanisms involving inhibition of the NLRP3/Caspase-1 signaling pathway (78). In a chronic-prostatitis rat model and a pyroptosis cell model, ursolic acid reduced inflammation by regulating the NLRP3 inflammasome-mediated Caspase-1/GSDMD pathway, decreasing ROS levels and inhibiting p-NF-κB protein expression *in vitro* and *in vivo*, and reversing the elevation of IL-1β and IL-18 (79). A human single-center cross-sectional study provides limited evidence regarding NLRP3 as a severity-associated biomarker rather than evidence for treatment efficacy. The study found higher NLRP3 levels in expressed prostatic secretions (EPS) from patients with CP/CPPS than from BPH and healthy control groups, together with positive associations with Bergman inflammation grade and symptom scores. Its ROC analyses compared adjacent Bergman grades rather than CP/CPPS with non-CP/CPPS controls (80). The reported ≥55.25 ng/mL cutoff should therefore be interpreted as an exploratory severity-stratification threshold rather than a diagnostic threshold. EPS NLRP3 may be a candidate severity-associated biomarker, but external validation is required before diagnostic or therapeutic use (**Table 1**).

### Candidate strategies targeting chemokine receptors

Chemokine signaling has emerged as a candidate therapeutic direction. In an EAP mouse model, a CXCR7 agonist significantly alleviated pain and prostatic inflammation by restoring the Th17/Treg balance and inhibiting the TGFβ/SMAD pathway, providing a new approach for targeting chemokine receptors for immune modulation (81). In an EAP mouse model, Ningmitai capsule treatment downregulated the pain-related factor CCL2 and inhibited NF-κB and STAT3 inflammatory pathways, thereby relieving pelvic pain (82). In a single-arm before-and-after study of 60 patients with CP/CPPS, of whom 52 completed treatment, low-intensity extracorporeal shockwave therapy was associated with symptom improvement and decreased TNF-α levels in seminal plasma, with efficacy possibly related to modulating the local chemokine environment (83). In a randomized study of 180 patients with type IIIb chronic prostatitis, combined tamsulosin and terazosin treatment reduced macrophage inflammatory protein-1α (MIP-1α) and MIP-2 levels in prostatic fluid and improved symptoms (84). Together, these findings nominate chemokine signaling as a candidate target but do not establish the efficacy of selective chemokine-receptor modulation in human CP/CPPS.

### Candidate strategies targeting the gut microbiota and metabolites

In a rat study combining EAP with chronic unpredictable mild stress, the combined-model group showed the greatest inflammatory changes together with distinct gut-microbial and predicted metabolic profiles, linking dysbiosis to prostatitis-associated depressive-like behavior (85). In a cross-sectional study of 53 men (22 with chronic-prostatitis symptoms and 31 asymptomatic controls), vaginal-microbiota-associated species were detected more often in controls, but the between-group differences were not statistically significant; the proposed protective role is therefore hypothesis-generating (86). Gut-ecology modulation has therefore been explored as a candidate systemic strategy. In EAP mice and complementary *in vitro* assays, dietary docosahexaenoic acid (DHA) attenuated disease severity and suppressed Th17 cell production by activating peroxisome proliferator-activated receptor γ (PPARγ) and thereby inhibiting the NF-κB/IL-17A pathway (87). In EAP mice, intraperitoneal sodium butyrate treatment reduced pelvic pain and prostatic inflammation and attenuated oxidative stress by activating the Nrf2/HO-1 signaling pathway, in turn inhibiting NLRP3 inflammasome activation (88). In an EAP mouse intervention study supplemented by gut-microbiota profiling in patients, astaxanthin reduced inflammation and pelvic tactile hypersensitivity in mice while increasing intestinal Akkermansia muciniphila, serum acetate, and gut-barrier integrity; the patient component was observational and did not evaluate astaxanthin treatment (89). In a high-fat-diet-induced obesity rat model with prostate hyperplasia and inflammatory injury, zinc supplementation reduced pro-inflammatory factor levels in prostatic tissue, with effects related to regulating zinc-transporter expression and inhibiting the JAK1/STAT3 inflammatory pathway (90). A total tanshinones extract of Salvia miltiorrhiza and its main component tanshinone IIA alleviate pain and inflammation in EAP rats by restoring gut-microbiota composition, lowering serum lipopolysaccharide levels, and downregulating TLR4-pathway expression; fecal microbiota transplantation experiments directly confirm a mediating role of the gut microbiota in this process (91). One randomized placebo-controlled clinical study of 124 patients with type IIIa prostatitis and concomitant irritable bowel syndrome reported that rifaximin combined with DSF probiotics improved urogenital and gastrointestinal symptoms and altered seminal-plasma inflammatory markers, providing subgroup-specific human evidence (92). In a mainstream-smoke-exposed mouse model and cigarette-smoke-extract-treated BPH-1 cells, Akkermansia muciniphila or its extracellular vesicles showed anti-inflammatory and anti-fibrotic effects, providing indirect preclinical evidence (93). In a carrageenan-induced CNP rat model, rapeseed bee pollen altered the gut microbiota, reduced pro-inflammatory cytokine expression, and improved prostate pathology, providing a basis for developing natural-product-based microbiota regulators (94).

### Local drug-delivery systems and physical therapies

To improve therapeutic efficiency and reduce systemic side effects, novel drug-delivery systems are being developed for local targeted treatment of prostatitis. In cell assays and a CNP rat model, a rectally administered hyaluronic acid/lactoferrin-modified emodin nanoparticle gel (HA/LF@EMO-NPs Gel) was shown to cross the blood–prostate barrier and accumulate within prostatic acini. After uptake by macrophages, the system modulates the TLR4/NF-κB pathway and exerts anti-inflammatory, antioxidant, and anti-fibrotic effects, highlighting the potential of nanoparticle-based rectal gel delivery for CP/CPPS treatment (95). In *in vitro* formulation studies and rat prostatitis experiments, another rectal gel combined thermotherapy with local delivery of Chrysanthemum indicum extract using a phase-change microemulsion. The gel provided sustained local release of heat and bioactive compounds. In rats, it enhanced drug accumulation in prostatic tissue, reduced the prostatic index, and suppressed inflammatory cytokines, suggesting synergistic benefits from local thermotherapy and targeted phytomedicine delivery (96). These preclinical delivery systems are designed to deliver therapeutic agents to the prostate, maximizing local drug concentration while minimizing impact on other tissues, representing a precision-medicine direction in CP/CPPS treatment. Low-intensity pulsed ultrasound (LIPUS) at an intensity of 1.0 W/cm² for 4 weeks showed the greatest efficacy among the tested regimens in a carrageenan-induced prostatitis rat model, significantly relieving pelvic pain and reducing prostatic inflammation scores, with mechanisms involving regulation of the Th17/Treg balance and downregulation of the IL-1β/IL1R1/MyD88 signaling pathway (97). In a hormone-induced CP/CPPS rat model, radiofrequency hyperthermia reduced pro-inflammatory cytokines and improved inflammation by downregulating HMGB1 expression and inhibiting the TLR4-NF-κB pathway, without significant damage to adjacent organs, supporting further preclinical evaluation of this physical approach (98).

### Chinese herbal formulas and natural products

Many natural products and Chinese herbal formulas intervene in the immunometabolic and cell-death networks of CP/CPPS through a multi-component, multi-target, multi-pathway mode of action. In network-pharmacology analyses and a CP/CPPS rat model, Qianlie Jindan tablets were associated with reduced IL-6/STAT3 signaling, Th17 differentiation, CD3+ T-cell infiltration, and ROS expression, together with improved prostate pathology (99). In a prostate-antigen-induced autoimmune chronic-prostatitis rat model and complementary *in vitro* assays, total flavonoids from Hosta plantaginea (Lam.) Aschers flowers showed anti-inflammatory activity by inhibiting the phosphorylated protein expression of multiple signaling pathways including NF-κB, MAPKs, JAK-STAT, and PI3K-Akt (100). In an EAP-induced inflammation-associated BPH rat model, Danzhi qing’e decoction reduced prostate enlargement and inflammatory macrophage infiltration; in an M2-macrophage-conditioned BPH-1 cell system, its active components tanshinone IIA and bakuchiol inhibited epithelial proliferation and ERK1/2 activation (101). In a CNP rat model, Qinghua decoction modulated inflammatory cytokines and the CgA/NGF/TrkA neuroendocrine-inflammatory signaling pathway (102). In network-pharmacology analyses combined with an Escherichia coli-induced bacterial-prostatitis experiment, Polygonum capitatum affected the PI3K-AKT pathway (103). Together, these studies offer mechanistic hypotheses and leads for further validation rather than evidence of clinical efficacy in human CP/CPPS. Cucurbitacin B, isolated from the fruit of Cucumis prophetarum, significantly ameliorates carrageenan-induced prostatitis in rats, lowers the prostatic index, and mitigates histopathological damage, with anti-inflammatory effects partly attributed to inhibiting neutrophil and macrophage infiltration into prostatic tissue (104). In a prostate-antigen-induced autoimmune chronic-prostatitis rat model, the polarity fractions of H. plantaginea flowers improved prostate pathology, with mechanisms involving inhibition of phosphorylated-protein expression in NF-κB, MAPKs, JAK-STAT, and PI3K-Akt signaling pathways (105).

### Neuromodulation adjunctive therapy and pain-signaling intervention

For the core symptom of CP/CPPS, chronic pelvic pain, directly modulating neuro-immune interactions and pain pathways is important in addition to anti-inflammation. In a complete-Freund’s-adjuvant-induced CPPS rat model, electroacupuncture modulated PI3K-AKT/JAK-STAT-related gene expression in the spinal dorsal horn and reduced inflammatory and pain responses (106). In LPS-stimulated RWPE-1 cells and an EAP mouse model, sertraline suppressed epithelial-cell proliferation and ROS generation, bound to and downregulated TRPV1, and reduced pain responses and prostatic inflammation, supporting further preclinical investigation of sertraline as an anti-inflammatory and analgesic approach (107). In a retrospective study of 105 patients with chronic prostatitis, transcutaneous electrical nerve stimulation combined with levofloxacin and tamsulosin was associated with greater reductions in pain and inflammatory markers than drug therapy alone, without an observed increase in safety events (108). In an RWPE-1 cell system and a hormone-induced rat prostatitis model, cannabidiol suppressed the TLR4/NF-κB pathway through CB2-receptor activation and produced dose-dependent analgesic effects associated with TRPV1 activation and desensitization, supporting preclinical investigation of both immune and pain pathways (109). An isolated-tissue study found that a pollen extract reduced LPS-induced IL-6 and TNF-α expression, while in silico analyses suggested interactions of catechin and chlorogenic acid with TRPV1; these indirect findings require validation in CP/CPPS models (110). The common goal of these strategies is to simultaneously intervene in abnormal activation of the immune system and sensitization of the nervous system, providing a mechanism-driven, integrative therapeutic perspective for CP/CPPS, particularly for patients with significant pain symptoms.

## Evidence limitations and controversies

The evidence base summarized in this narrative review is heterogeneous. Most mechanistic and interventional findings derive from *in vitro* systems, EAP or CNP models, whereas direct human evidence is limited and is largely cross-sectional, observational, or based on small clinical studies. These models reproduce selected inflammatory and pain-related features but do not capture the etiologic, symptomatic, metabolic, and psychosocial heterogeneity of human CP/CPPS; findings from BPH, bacterial prostatitis, obesity-, hormone-, cigarette-smoke-, or injury-induced prostate models are even more indirect. Studies also differ in model induction, tissue compartment, immune markers, outcome measures, and intervention timing, which limits comparison and may contribute to apparently inconsistent findings, including the context-dependent roles of M2 macrophages, microbiota-derived metabolites, and inflammatory mediators. Longitudinal validation in well-phenotyped patients, replication across centers, and prospective trials are therefore required before candidate biomarkers, immune endotypes, or targeted interventions can be considered clinically established.

## Conclusion and outlook

This narrative review synthesizes recent advances in immune-related mechanisms of CP/CPPS across macrophage heterogeneity, NLRP3 inflammasome activation, immunometabolic reprogramming, chemokine networks, and neuro-immune crosstalk. Available evidence suggests that chronic inflammation and pain may arise from interactions among innate and adaptive immunity, cellular metabolism and cell death, and peripheral and central neural pathways. The M1/M2 framework may not fully capture disease-associated macrophage states; the NLRP3-IL-1β axis is a candidate link between tissue damage and Th17 responses; glycolytic and oxidative-stress-related changes may contribute to immune-cell activation; ferroptosis and pyroptosis may participate in inflammatory amplification; and interactions among mast cells, macrophages, and sensory neurons may help sustain pelvic pain.

Future research should prioritize translation from mechanistic hypotheses to clinically testable precision-medicine approaches. Single-cell and spatial multi-omics studies of well-characterized, adequately powered patient cohorts could map disease-associated immune-cell states and relate them to symptoms and treatment responses. Candidate biomarkers will require multicenter analytical and clinical validation before use in patient stratification. Therapeutic development should test whether multi-target approaches, local delivery systems, microbiota-directed interventions, or neuro-immune modulation improve clinically meaningful outcomes without unacceptable toxicity. Prospective umbrella or basket trials could evaluate candidate therapies in immune-phenotype-defined subgroups and determine whether mechanism-guided strategies outperform current symptom-based care.

## Glossary

5-HIAA: 5-hydroxyindoleacetic acid
ACSL4: Acyl-CoA synthetase long-chain family member 4
AhR: Aryl hydrocarbon receptor
AKT: Protein kinase B
ANXA1: Annexin A1
AP-1: Activator protein-1
AR: Androgen receptor
BPH: Benign prostatic hyperplasia
CB2: Cannabinoid receptor type 2
CCL2 (MCP-1): C-C motif chemokine ligand 2 (monocyte chemoattractant protein-1)
CCL5 (RANTES): C-C motif chemokine ligand 5 (regulated upon activation, normal T cell expressed and secreted)
CCL20: C-C motif chemokine ligand 20
CCR5: C-C chemokine receptor type 5
CD3/CD4/CD8/CD11b/CD44/CD45/CD74: Cluster of differentiation (immune-cell surface markers/receptors, numbered by antigen)
cGAS–STING: Cyclic GMP–AMP synthase–stimulator of interferon genes
CGRP: Calcitonin gene-related peptide
CgA: Chromogranin A
c-MYC: Cellular myelocytomatosis oncogene
CNP: Chronic non-bacterial prostatitis
CP/CPPS: Chronic prostatitis/chronic pelvic pain syndrome
CPP: Chronic pelvic pain
CPPs: Calciprotein particles
CXCL1/CXCL2/CXCL8/CXCL10 (IP-10)/CXCL12: C-X-C motif chemokine ligand 1/2/8/10/12
CXCR1/CXCR3/CXCR4/CXCR7: C-X-C chemokine receptor type 1/3/4/7
DAMPs: Damage-associated molecular patterns
DHA: Docosahexaenoic acid
DSF: De Simone Formulation (multistrain probiotic preparation)
dsHMGB1: Double-stranded high-mobility group box 1
EAP: Experimental autoimmune prostatitis
ERK1/2: Extracellular signal-regulated kinase 1/2
FoxO1: Forkhead box protein O1
FXR: Farnesoid X receptor
GPR43: G protein-coupled receptor 43
GPX4: Glutathione peroxidase 4
GSDMD: Gasdermin D
HA: Hyaluronic acid
HDAC6: Histone deacetylase 6
(Hif-1α): Hypoxia-inducible factor-1α
HMGB1: High-mobility group box 1
HO-1: Heme oxygenase-1
IGF1/IGF1R: Insulin-like growth factor 1/IGF1 receptor
IL-1β/IL-6/IL-8/IL-10/IL-10RA/IL-12/IL-12B/IL-17/IL-17A/IL-18: Interleukin-1β/6/8/10/10 receptor subunit α/12/12B/17/17A/18
IL1R1: Interleukin-1 receptor type 1
iNOS: Inducible nitric oxide synthase
IRF7 (Irf7): Interferon regulatory factor 7
JAK1/JAK2: Janus kinase 1/2
Keap1: Kelch-like ECH-associated protein 1
KCNN4: Potassium calcium-activated channel subfamily N member 4
LIPUS: Low-intensity pulsed ultrasound
MAPK(s): Mitogen-activated protein kinase(s)
MIF: Macrophage migration inhibitory factor
MIP-1α/MIP-2: Macrophage inflammatory protein-1α/-2
miR-203a-3p: MicroRNA-203a-3p
mMCP6/mMCP7: Mouse mast cell protease 6/7
mTOR/mTORC1: Mechanistic target of rapamycin/mTOR complex 1
mtDNA: Mitochondrial DNA
MyD88: Myeloid differentiation primary response 88
NF-κB: Nuclear factor kappa B
NFE2L2: Nuclear factor erythroid 2-like 2
NGF: Nerve growth factor
NIH-CPSI: National Institutes of Health Chronic Prostatitis Symptom Index
NLRP3: NLR family pyrin domain-containing protein 3
NMDA/NR1: N-methyl-D-aspartate (receptor)/NMDA receptor subunit 1
NOTCH1: Notch homolog 1
NOX4: NADPH oxidase 4
NQO1: NAD(P)H quinone dehydrogenase 1
Nrf2: Nuclear factor erythroid 2-related factor 2
P2X7R: P2X purinoceptor 7
PAR2/PARs: Protease-activated receptor 2/protease-activated receptors
PFKFB3: 6-phosphofructo-2-kinase/fructose-2, 6-biphosphatase 3
PI3K: Phosphoinositide 3-kinase
PKC-β: Protein kinase C-β
PPARγ: Peroxisome proliferator-activated receptor γ
Prdx5: Peroxiredoxin 5
RNA-seq: RNA sequencing
ROC: Receiver operating characteristic
RORγt: Retinoic acid receptor-related orphan receptor gamma t
ROS: Reactive oxygen species
SCFA(s): Short-chain fatty acid(s)
SerpinB2: Serine protease inhibitor B2
Sgk1: Serum and glucocorticoid-regulated kinase 1
SHP2: Src homology 2 domain-containing protein tyrosine phosphatase 2
SMAD: Sma- and Mad-related protein family
SP: Substance P
SREBP2: Sterol regulatory element-binding protein 2
STAT1/STAT3/STAT4/STAT6: Signal transducer and activator of transcription 1/3/4/6
TEMRA: Terminally differentiated effector memory T cells re-expressing CD45RA
TENS: Transcutaneous electrical nerve stimulation
TGF-β: Transforming growth factor-β
Th1/Th17: T helper 1/17 cell
TLR4: Toll-like receptor 4
TNF-α: Tumor necrosis factor-α
TRAF6: TNF receptor-associated factor 6
Tregs: Regulatory T cells
TrkA: Tropomyosin receptor kinase A
TRPV1/TRPV2: Transient receptor potential vanilloid 1/2
UCPPS: Urological chronic pelvic pain syndrome
xCT: Cystine/glutamate antiporter light-chain subunit (SLC7A11)
